# Effects of workbook training using editorials and newspaper articles in adults with preclinical stage of dementia

**DOI:** 10.1038/s41598-024-52873-z

**Published:** 2024-01-27

**Authors:** Sora Jin, Ji Hye Yoon, Duk L. Na

**Affiliations:** 1 Department of Speech Pathology and Audiology, Graduate School at Hallym University, Chuncheon, South Korea; 2 Division of Speech Pathology and Audiology, Research Institute of Audiology and Speech Pathology, Hallym University, Chuncheon, South Korea; 3 Department of Neurology, Samsung Medical Center, Sungkyunkwan University School of Medicine, Seoul, South Korea

**Keywords:** Health care, Neurology

## Abstract

Early detection and intervention in individuals in the pre-clinical stage of dementia are crucial. This study aimed to examine whether there are significant differences in (1) word retrieval, (2) subjective communication ability, (3) intervention satisfaction through the 'Fill-in-the-blanks in editorial and newspaper articles' training in patients with subjective cognitive decline and mild cognitive impairment corresponding to the pre-clinical stage of dementia. Ninety-nine patients (50 in the intervention group and 49 in the control group) aged 50–84 years were administered pre- and post-test after 6 weeks of intervention (30 sessions). Regarding word retrieval, there were significant intervention effects on confrontation naming, semantic fluency, and phonemic fluency. The majority of participants in the intervention group were highly satisfied with the training. In terms of intervention satisfaction, the majority of the participants in the intervention group showed high satisfaction with all the questions. This result confirmed the improvement of word retrieval ability through mass communication content-based 'Fill-in-the-blanks' training, and ultimately helps to provide a clinical basis for applying this intervention to prevent dementia.

## Introduction

According to Statistics Korea^1^, the number of elderly aged over 65 years was 7,685,000, accounting for 14.9% of the total population in the Republic of Korea, and thus became an aged society. With a rapid increase in Korea's elderly population in recent years, dementia, a representative degenerative disease of the elderly, is also emerging as a social issue. The most common cause of dementia is Alzheimer's disease (AD), which accounts for about 74.5% of all dementia^2^. The disease develops slowly causing deterioration of cognitive functions, including memory and progresses gradually. Therefore, controlling the progression of the disease after its onset is challenging. Therefore, early detection, intervention and classification of high-risk groups through evaluation of individuals in a mild pre-clinical stage before progressing into dementia of the Alzheimer's type (DAT) and to delay the progression of the disease through active intervention is crucial^3^.

Mild cognitive impairment (MCI), a stage between normal aging and dementia within the continuous spectrum of cognitive aging, is a group of diseases with an estimated 8–16% annual rate of developing dementia^4^. Clinically, although their overall cognitive function and activities of daily living (ADL) are normal, they are characterized by impaired memory and other cognitive functions (including language, executive function, and visuospatial function). The National Institute on Aging-Alzheimer's Association has suggested that MCI caused by Alzheimer’s disease is associated with two factors: (1) beta-amyloid (Aβ) biomarkers (e.g. positive Pittsburgh compound B or cerebrospinal fluid (CSF) Aβ42) and (2) neurodegenerative biomarkers (e.g. CSF tau, fluorodeoxyglucose positron emission tomography, or structural magnetic resonance imaging). The likelihood of MCI is high if both factors are positive, moderate if either is positive, and low if both are negative^5^. These biomarkers are used in clinical settings for diagnostic purposes, even though evidence for the rational use is incomplete. Recently, a multidisciplinary group of experts have been attempting a rigorous evidence-to-decision approach based on a systematic literature review and consensus procedure. The process aims to define the diagnostic process required for the rational use of biomarkers to provide the etiological definition of MCI^6–9^.

As a standard to classify MCI, Petersen^10^ described MCI as an amnestic MCI single domain (aMCI-single), if only memory is impaired according to an abnormality in the memory domain, and an amnestic MCI multiple (aMCI-multiple), if there is damage to one or more other cognitive domains, including impairment in the memory domain. Similarly, in the non-amnestic MCI single domain (naMCI-single), only one domain is damaged in other cognitive domains without memory impairment. In the non-amnestic (naMCI-multiple) category, there is damage to two or more other cognitive domains without memory impairment. Among them, aMCI has the highest possibility of developing Alzheimer’s dementia^5^. In addition, recently, various clinical terms including the word or meaning of “subjective” are used as keywords to define the intermediate stage between normal aging and MCI^11^. Among such terms, subjective cognitive decline (SCD), is said to have occurred if the performance on a standardized cognitive test is within the normal range, but cognitive function continues to deteriorate^12^. Previously, in terms of normal cognitive function, individuals with SCD have been known to react sensitively to age-related changes. However, a recent study observed functional changes in the brain^13^, and according to a follow-up study, the annual rate of progression from SCD to MCI reached 6.6%^14^. Moreover, SCD has been suggested as a prognostic marker for MCI and dementia^15^. Therefore, early detection and intervention at the SCD stage before MCI is crucial.

Several previous studies have revealed that naming disorders appear at the earliest stage of the disease, along with memory problems in DAT^16–18^. Accordingly, a specific linguistic change pattern has become an important indicator for the early diagnosis of DAT. In the continuum of cognitive aging, in the case of MCI, research results on naming ability are contradictory^19^, but as semantic knowledge gradually decreases, word-finding difficulties appear^20,21^. Although SCD has a better confrontation naming ability than MCI^22^, it frequently experiences a tip-of-the-tongue (TOT) phenomenon, in which people get temporarily interrupted when trying to produce the names of well-known words^23,24^. In studies related to naming interventions, semantic feature analysis (SFA) has been mainly used to intervene in noun naming. For DAT, the intervention was performed using a computer program where the existing SFA was modified and supplemented with the noun-naming intervention, which provided both semantic features and phonological cues^25^. SFA intervention using noun familiarity^26^ improved the correct response rate of the target word.

However, in MCI studies related to naming interventions were limited. In a study that conducted an SFA intervention for middle-aged and older adults in the community who subjectively complained of a decrease in naming ability and confirmed a change in naming ability, improvement was observed in the Korean-Boston Naming Test (K-BNT) and in naming items not included in the intervention list after the intervention^27^. However, the stimuli used in prior studies are mostly familiar words or concrete nouns used in daily life and are mostly words used in research or evaluation situations. Moreover, since the above studies selected a treatment method using pictures or drawings of objects for naming interventions, there was a limitation of using only concrete nouns that can be implemented as specific pictures as tasks. Therefore, including low-frequency or abstract words used in daily life is challenging. However, to effectively mediate participants who show a slight decrease in name recognition ability, such as patients in the preclinical stage of dementia, high-level tasks including low-frequency words, abstract words, and Chinese-derivative words’ are required.

Herein, we devised a workbook training program using the latest editorials and newspaper articles by supplementing the limitations of previous studies and considering the quality of life and functional communication in terms of communication and word retrieval in real life. Newspaper articles and editorials are facts and opinions that reflect the times, bridge gaps in communication and activities in the social context, and enable members to access society economically and intimately. Moreover, in addition to high-frequency words, these articles also contain low-frequency words, including abstract words and Chinese-derivative words. This can be widely used to improve the ability of adults with a mild decline in ability due to the preclinical stage of dementia. Therefore, for patients with the preclinical stage of dementia, workbook training using editorial and newspaper articles was conducted in the form of self-intervention five times a week for six weeks, and the effects were confirmed through a pre-post comparison in terms of (1) word retrieval, (2) subjective assessment of communication ability, and (3) intervention satisfaction.

## Methods

### Participants

Using the F-test using the G*power 3.1.9.2 program, we found that a sample size of more than 92 people was an appropriate number of participants at effect size 0.15, significance level 0.05, and power 80%. The sample was consecutively selected among patients who visited the neurology department of the general hospital. This study involved 99 adults (50 in the intervention group and 49 in the control group) aged 50–84 years who were in the preclinical stage of dementia. Diagnosis was perfomed by a neurologist based on a neurological examination and a review of the patients’ medical history. All participants included met the criteria for selection and provided informed consent. The recruited participants were assigned a number according to random ordering (randomized control trial design). Those assigned odd numbers were included in the intervention group and even numbers were included in the control group. All participants participated in the entire process with a 0% drop-out rate.

The 99 participants included 50 adults diagnosed with SCD (25 each in the intervention and control group) and 49 adults diagnosed with MCI (25 patients in the intervention group and 24 patients in the control group). The MCI group included 39 aMCI and 10 naMCI patients. As the preliminary step for the study, we compared specific characteristics (age, education, and SGDS) of the SCD and MCI groups. An independent sample *t* test was conducted to assess differences in the characteristics between the SCD and MCI groups, and there were no significant differences in age (t = − 0.551, p = 0.583), number of years of education (t = 0.247, p = 0.805), and SGDS scores (t = − 0.117, p = 0.907). In addition, independent sample t-tests were conducted to investigate the differences between the intervention and control groups in the SCD and MCI groups. We found that there were no significant differences in the demographic information of the intervention and control groups in the SCD and MCI groups. These preliminary results are presented in the Appendix. Based on this homogeneity, we combined the two groups and termed the group the ‘preclinical stage of Alzheimer's type dementia.’ Between the experimental and control groups, there were no significant differences in age (t = 0.646, p = 0.520), years of education (t = − 0.171, p = 0.864), K-MMSE (t = − 1.552, p = 0.124), or SGDS (t = 0.040, p = 0.969) scores. Data on participant characteristics are presented in Table 1.

**Table 1:** Participants’ characteristics.

Characteristic	Intervention group (N = 50)	Control group (N = 49)	T
Age (years)	72.22 (6.92)	71.32 (6.82)	0.646
Education (years)	13.86 (2.91)	13.95 (2.85)	− 0.171
K-MMSE	27.78 (1.99)	28.38 (1.90)	− 1.552
SGDS	3.00 (2.46)	2.97 (2.66)	0.040

*K*-*MMSE* Korean version of mini-mental state examination, *SGDS* short form geriatric depression scale.

### Intervention materials

The ‘workbook using editorial and newspaper articles’ training is for middle-aged adults who are in the pre-clinical stage with mild symptoms before AD progression. It is designed to improve word retrieval by utilizing context while gaining knowledge about life through the latest editorials and newspaper articles. Hence, it is a self-intervention training program developed to improve functional communication and quality of life in terms of word retrieval. The main training format of this program was the ‘fill-in-the-blanks’ task, which was borrowed from the format of the sentence completion task, one of the naming tasks of the Korean-Western Aphasia Battery(K-WAB)^28^ (e.g. roses are red and forsythia is____?). For the fill-in-the-blanks task, the researcher selected major articles and editorials on the front page of a newspaper among articles published in newspapers within the last year. It was created by abbreviating articles between 400 and 600 letters and making blanks by selecting 10 keywords per passage. The difficulty of the words in the blanks was divided into thirds according to the standard of ‘Contemporary Korean Use Frequency Survey 2’ of the National Institute of the Korean Language^29^, and words with a frequency of 66% or less were selected as middle and low frequencies. The text source was indicated at the bottom of each task. The workbook consisted of material for 30 days (6 weeks, 5 days a week), and two fingerprints (one editorial and newspaper article each) were included in the one-day supply. The total number of words in the workbook was 600. The first chapter of the workbook is created by printing text including the blanks to be filled in by the participants themselves. In the second chapter, the initial consonant and semantic cues of the target words corresponding to each blank are presented for naming using cues as much as possible in a situation where self-intervention training cannot help in filling in the blanks. Definitions from the Korean Standard Dictionary^30^ were used as semantic cues. After creating the workbook, a Likert scale ranging from 1 to 5 points (1 = Very inappropriate. 2 = Not appropriate, 3 = Moderate, 4 = Appropriate, 5 = Very appropriate) was used to assess the appropriateness of the content and tasks for improving word extraction ability. Three certified speech-language pathologists (SLPs) with doctorate degrees in speech-language pathology evaluated eight items of the validity of the workbook. The mean value of all items was greater than the 4.3 point, signifying the content and tasks were appropriate.

Two naming tasks (letter completion according to semantic category and word generation using initial consonants) were used as additional training tasks. In the letter completion task, a letter is completed by looking at the phoneme presented after a semantic category (for example, This is a word related to ‘animal’. Complete the letters). In the word generation task using the initial consonant, two consecutive consonants are presented initially and using these consonants, the participants need to write down ≥ 10 meaningful words (e.g. Write 10 words that start with ‘’). The procedure for performing the task was presented at the beginning of the workbook. The researcher reviewed the procedure for approximately 5 to 10 min, demonstrated it orally, and explained how to solve the workbook to the participants, to confirm the participants’ familiarity with the training method.

### Intervention procedures

This intervention was conducted as a self-intervention method for 30 sessions, five days per week over 6 weeks. The workbook was handed out by the researcher directly to the intervention group that had completed the pre-test. Initially, the participants had 5 to 10 min to review, demonstrate, and explain orally the method to solve the workbook. Their level of familiarity with the training method was also confirmed. During the workbook implementation period, the following instructions were provided: “(1) Please complete the workbook regularly from Monday to Friday. If you cannot solve it for personal reasons, please extend it to weekends. (2) We will text you daily to ensure that you have completed your workbook. Kindly reply. (3) If you are unsure how to solve the workbook, you can contact us by phone at any time. (4) Please be honest in terms of solving the workbook.” For effective self-intervention, the researcher contacted the participants using text messages and phones every day during the training period. When the task was not completed within a set time, a text message was sent to encourage the participants to complete the task. The average task performance time per session was 40–50 min.

The workbook was used as follows: First, the participants were asked to read the editorial and newspaper articles aloud on the right page and write the appropriate words in the blanks. Unknown answers were left blank. On the next page, the semantic and phonemic cues of the target word were presented, for filling in the blanks. Third, the intervention group was asked to complete the letter completion task and initial consonant word task of the subtasks on the right page. For the letter completion and initial consonant word tasks, a separate correct answer sheet was provided for filling in the correct answer with a different colored ballpoint pen for the wrong questions. Subsequently, by presenting the correct answer in the last chapter, the participant repeated reading the text aloud, returned to the fill-in-the-blanks task in the first chapter, and remembered the answer without looking at the correct answer to fill in the remaining blanks. After completing the workbook, the intervention group visited the hospital within a week to submit the workbook and undergo a post-test, which was conducted only for the intervention group participants, who completed the workbook. In addition, during the post-test, feedback on the benefits and challenges associated with the workbook was obtained. The control group participated only in the pre-post test without self-intervention training. Approval was granted by the Ethics Committee of Institutional Review Board of Samsung Medical Center (#2020-03-017-003).Confirms that all experiments were performed in accordance with relevant named guidelines and regulations. The procedures used in this study adhere to the tenets of the Declaration of Helsinki.

### Instruments

With respect to the inclusion and selection of participants, the following criteria and screening tests were used to select adults with the preclinical stage of dementia. The SCD participants (1) had no history of mental and neurological disease on the health screening questionnaire^31^, (2) complained of memory impairment and scored ≥ 25 in the Memory Age associated Complaint-Questionnaire^32^, (3) had scores within or greater than the sixteeth percentile on the Korean-Mini Mental State Examination (K-MMSE)^33^ and Seoul Neuropsychological Screening Battery (SNSB-II)^34^ (as a standardized test designed to comprehensively evaluate the five cognitive domains: Attention, Language & Related Functions, Visuospatial Functions, Memory, Frontal/Executive Function), (4) had no depression with a score ≤ 8 on the Short Form Geriatric Depression Scale (SGDS)^35^, and (5) had recived education above middle school level. The MCI participants were diagnosed by a neurologist using the criteria of Petersen^10^ based on a neurological examination and medical history. The detailed criteria are as follows: The MCI participants (1) scored 0.5 on the clinical dementia rating (CDR), (2) had the subjective memory impairment complaint by the person or guardian, (3) scored within or greater than the sixteeth percentile on K-MMSE, and had normal overall cognitive function (4) had scores within or greater than the sixteeth percentile based on age and years of education of the SNSB-II^34^ as objective memory impairment, but had no difficulty in performing daily-life activities by scoring < 8 on the Seoul-Instrumental Activities of Daily Living; S-IADL)^36^, (5) did not correspond to the criteria for dementia, and (6) were with above middle school education.

With respect to the pre-post test, tests were conducted involving (1) word retrieval, (2) subjective communication ability, and (3) intervention satisfaction to investigate the effects of the intervention. In the pre-test, the baseline of word retrieval and subjective communication were measured. The post-test was the same as the pre-test, but a satisfaction questionnaire was added to the intervention group to confirm satisfaction with the intervention. Among these tests, the test of subjective communication ability was a self-report questionnaire. Thus, both the pre- and post-questionnaires were provided as home assignments. The pre-test questionnaire was completed by the participantt at home on the day of the pre-test, and the participant was then asked to take a picture of the questionnaire and send it to the researcher. The post-test questionnaire was completed by the participantthrough a telephone call with the researcher the day before the post-test, and the participant was asked to present the questionnaire on the day of the post-test. Except for the participant communication ability test, the other tests were conducted individually in an independent space and in total took approximately 45 min. The participants were fully informed about the test task before the start of the test. The researcher sat with the participants and recorded their responses.

The word retrieval was evaluated by dividing it into a confrontation naming task, generative naming task, and fill-in-the-blanks task, considering the task type. A short form of the Korean-Boston Naming Test (S-K-BNT)^37^ and a difficult naming test (DNT)^38^ were conducted as confrontation-naming tasks. The S-K-BNT is a standardized test and DNT is currently undergoing standardization and will be published. In a previous study^39^, when comparing the characteristics of confrontation naming task according to the frequency of words in the normal elderly group and the MCI group, the frequency effect of words was higher in DNT than in K-BNT. The previous study has also shown that DNT showed statistically high correlation with K-MMSE and K-BNT. To measure the number of correct responses and reaction time, both naming tests were performed using the E-prime 3.0 program and tested through a 15.6-inch screen. The participants’ responses were recorded using E-prime 3.0. The face-to-face naming test was analyzed in terms of the 'number of correct responses' and 'reaction time'. Regarding the number of correct responses, if an accurate target word was voluntarily produced based on the procedure manual, it was regarded as a correct response and one point was awarded. If an incorrect word was produced or a correct response was produced for an item that had already been provided in the past, it was considered an incorrect response. For reaction time, Praat, a speech analysis program was used and only when the correct target word was produced, the time from the moment the picture was presented, to the moment the participant responded verbally was analyzed.

For the generative naming task, semantic and phonemic fluency tasks were measured. These fluency tasks are sub-tests to assess the frontal lobe/executive function of the SNSB-II^34^. For the semantic fluency task, the semantic categories of 'animal' and 'supermarket' were used^40^. All participants’ responses were recorded. Before starting the test, the researcher instructed as follows: “When I say ‘Start.’ quickly say as many animal names that come to your mind. You have to keep saying the names until I say, ‘Stop.’ The time limit is 1 min. Are you ready? Start.” All participants’ responses were recorded on a test record sheet. In the semantic fluency task, one point was awarded for a correct response only when words belonging to the relevant category (animals/supermarkets) were correctly spoken among the number of words spoken in one minute. Repeated words or those that did not fall into the particular category were excluded from the score. When words from the superordinate and subordinate concepts were produced together (e.g. bird, goose, cuckoo, dove), only words from the specific subordinate concept (e.g. goose, cuckoo, dove) were included in the score, and words from the superordinate concept (e.g. bird) were excluded. In the phonemic fluency task, were evaluated. Before starting the test, the researcher instructed the participants as follows: “This time, if I tell you a certain letter, please say as many words that start with that letter. For example, if I say, ‘Tell me a word that starts with the letter ∟,’ you can answer something like . Subsequently, to verify whether the participants understood the instructions, they were asked to provide one or two examples. The researcher instructed as follows: “If you are ready, we will start. Say as many words that start with the letter ‘’ as quickly as possible”. After the “start” instruction, the test began. All participants’ responses were recorded on a test record sheet. Correct responses were awarded one point. When the participantrepeated a word or produced a word that did not start with the corresponding phoneme or produced a proper noun such as a person's name or city name, the score was not awarded. For derivative words, only the first response was considered correct.

The Probe test is a ‘fill-in-the-blank’ task and has the same format as the workbook. When the content validity was evaluated for three SLPs who obtained doctorate degrees in speech-language pathology, the mean value of all items was greater than the 4.3 point. For the fill-in-the-blanks test, two texts (one editorial and one newspaper article) that were not included in the workbook were presented. One text contained 10 fill-in-the-blanks. Before starting the test, the researcher instructed as follows: “There are two texts, please solve as hard as you can. Unsolved problems can be left.” Considering individual performance, no time limit was set, and the average task execution time was approximately 5–15 min. The synonyms of the target words to be filled in the blanks were regarded as correct answers.

For measuring subjective communication ability, the geriatric index of communicative ability (GICA)^41^, a standardized test tool, was used. The GICA consists of 18 items; however, in this study, only 12 items were used, excluding the auditory and voice areas that were not related to the intervention. The analysis used a Likert scale of 1–5 points, with a maximum score of 60 points; a higher score meant that there was no inconvenience in communication and it was maintained.

For measuring satisfaction with the intervention, the satisfaction questionnaire was administered to the intervention group. Based on the feedback of the workbook users, the researcher made 7 items and used them on a 1–5 point scale (‘Very Much, Somewhat, Undecided, Not Really, Not at All’). The researcher instructed as follows, “Please select the score that corresponds to the items asking about your satisfaction with the workbook training over the past 6 weeks.” For the satisfaction questionnaire, the maximum score was 35 points, with higher scores indicating higher satisfaction with the intervention. The detailed items are listed in Table 2.

**Table 2:** Satisfaction questionnaire items.

Items	
1.	I am generally satisfied with the workbook homework
2.	My self-confidence has improved
3.	The will to live has increased
4.	Positive thoughts (hope) and emotions (happiness) increased
5.	It gave me an opportunity to check about the problem of not remembering words by myself
6.	I have the motivation to pursue other studies
7.	I think it has helped prevent dementia

### Statistics

SPSS (version 25.0) was used for the statistical analyses of the data. A repeated two-way analysis of variance (ANOVA) was performed to examine whether there was a significant intervention effect (p < 0.05) in terms of word retrieval and subjective appeal according to the group before and after the intervention. For the post-hoc analysis according to the main effect, a paired-sample t-test of the corresponding sample was performed. To control for type I error across multiple testing, the significance probability was corrected with Bonferroni correction. Since a paired t-test performed twice for each variable, the significance level has been revised to 0.025.

### Ethics approval

This study was performed in line with the principles of the Declaration of Helsinki. Approval was granted by the Ethics Committee of Institutional Review Board of Samsung Medical Center (#2020-03-017-003).

### Consent to participate

All the participants provided informed consent before participation.

## Results

### Changes in naming ability according to intervention

The repeated two-way ANOVA revealed that there was an interaction effect (F_(1, 97)_ = 6.567, p = 0.012) according to the group × test period in the number of correct responses to the S-K-BNT, and the main effect (F_(1, 97)_ = 5.086, p = 0.026) according to the test period occurred, and there were no differences between groups (F_(1, 97)_ = 0.643, p = 0.425). Regarding the paired t-test for post-test according to the test period, the post-score of the intervention group significantly increased compared to the pre-score of the S-K-BNT (t = − 3.000, p = 0.004). Since there was no significant difference in the control group (t = 0.260, p = 0.796), the effect was only observed in the intervention group. The number of correct responses of DNT had no interaction effect (F_(1, 97)_ = 1.604, p = 0.208) according to the group × test period, and there was no difference between groups (F_(1, 97)_ = 0.478, p = 0.491). However, the main effect according to the test period (F_(1, 97)_ = 26.251, p < 0.001) was significant. As a result of the paired sample t-test for the test period, both the intervention group (t = − 4.229, p < 0.001) and the control group (t = − 2.954, p = 0.005) significantly increased the post-score compared to the pre-score. The reaction time of DNT had no interaction effect (F_(1, 97)_ = 0.037, p = 0.847) according to the group × test period, and there was no difference between groups (F_(1, 97)_ = 1.371, p = 0.244). However, the main effect according to the test period was significant (F_(1, 97)_ = 42.581, p < 0.001). As a result of the paired sample t-test for the test period, both the intervention group (t = 4.114, p < 0.001) and the control group (t = 5.287, p < 0.001) significantly increased the post-score compared to the pre-score. In the reaction time of S-K-BNT (F_(1, 97)_ = 0.583, p = 0.447), there was no interaction effect according to the group × test period, and there was no difference between groups (F_(1, 97)_ = 0.104, p = 0.748). However, the main effect according to the test period was significant (F_(1, 97)_ = 9.161, p = 0.003). As a result of the paired-samples t-test for the test period, there was no effect in the intervention group (t = 1.560, p = 0.125), and an effect was found only in the control group (t = 2.757, p = 0.008).

In the semantic fluency task on generative naming, an interaction effect (F(1, 97) = 17.214, p < 0.001) according to the group × test period and a main effect (F(1, 97) = 4.10, p = 0.046) according to the test period were observed; however, there was no difference between the groups (F(1, 97) = 1.729, p = 0.192). In the paired t-test for post-analysis according to the test period, the intervention group showed a significant increase in the post-score compared with the pre-score of the semantic fluency task (t = − 4.916, p < 0.001). However, there was no significant difference in the control group (t = 1.359, p = 0.180). Moreover, in the phonemic fluency task, an interaction effect (F(1, 97) = 43.755, p < 0.001) according to the group × test period and a main effect (F(1, 97) = 25.369, p < 0.001) according to the test period were observed. However, there was no difference between the groups (F(1, 97) = 3.124, p = 0.080). In the paired t-test for post-analysis according to the test period, the intervention group showed a significant increase in the post-score compared to the pre-score of the phonemic fluency task (t = − 7.480, p < 0.001). However, there was no significant difference in the control group (t = 1.265, p = 0.212).

In the fill-in-the-blank task analysis, which is a probe test, an interaction effect (F_(1, 97)_ = 13.198, p < 0.001) according to the group × test period and a main effect (F_(1, 97)_ = 42.340, p < 0.001) according to the test period were observed; however, there was no difference between the groups (F(_(1, 97)_ = 0.463, p = 0.498). In the paired t-test for post-analysis according to the test period, the intervention group showed a significant increase in the post-score compared to the pre-score of the prove test (t = -6.771, p =  < 0.001). There was no significant difference in the control group (t = 2.175, p = 0.035) (adjusted significance level = 0.025). Table 3 presents the number of correct responses and reaction times for each group according to the word retrieval task and test period.

**Table 3:** Comparison of word retrieval ability and old age communication ability scale according to pre-post test.

		Intervention group (N = 50)		Control group (N = 49)		Effect comparison	
		Pre	Post	Pre	Post		F
Confrontation naming task	S-K-BNT, number of correct responses (max = 15)	11.26 (1.96)	11.90 (1.72)	11.87 (1.83)	11.83 (1.82)	Between groups	0.643
						Test period	5.086**
						Group x test period	6.567*
	DNT, number of correct responses(max = 30)	19.00 (5.00)	20.42 (4.66)	19.97 (5.53)	20.83 (5.33)	Between groups	0.478
						Test period	26.251***
						Group x test period	1.604
	S-K-BNT, reaction time	2.13823 (0.60)	1.95265 (0.67)	2.16260 (0.81)	1.85175 (0.75)	Between groups	0.104
						Test period	9.161**
						Group x test period	0.583
	DNT reaction time	3.36509 (1.14)	2.70206 (0.61)	3.19676 (1.15)	2.49330 (0.79)	Between groups	1.371
						Test period	9.161**
						Group x test period	0.037
Generative naming task	Semantic fluency	28.32 (9.72)	32.00 (9.20)	33.22 (9.68)	21.95 (10.04)	Between groups	1.729
						Test period	4.10*
						Group x test period	17.214***
	Phonemic fluency	30.62 (13.43)	41.62 (12.57)	32.40 (14.72)	30.91 (12.75)	Between groups	3.124
						Test period	25.369***
						Group x test period	43.755***
Fill-in-the-blank task	Probe test	10.04 (3.97)	12.92 (3.46)	10.57 (4.33)	11.38 (3.86)	Between groups	0.463
						Test period	42.340***
						Group x test period	13.198***
Geriatric index of communicative ability (max = 60)		41.16 (7.37)	40.50 (7.65)	42.28 (8.20)	43.04 (7.59)	Between groups	1.586
						Test period	0.008
						Group x test period	1.771

*S-K*-*BNT* short-Korean-boston naming test, *DNT* difficult naming test.

***Significant difference between Intervention and Control group with *p < 0.05, ** p < 0.01, *** p < 0.001.

### Changes in subjective communication ability scales according to intervention

In the repeated two-way ANOVA to examine whether there was a significant difference in the GICA according to the intervention, the interaction effect according to the group × test period (F_(1, 97)_ = 1.771, p = 0.186), the difference between groups (F_(1, 97)_ = 1.586, p = 0.211), and the test period (F_(1, 97)_ = 0.008, p = 0.929), there were no main effects. Table 3 shows the descriptive statistical results of the GICA according to intervention.

### Results on satisfaction survey

Regarding the intervention satisfaction questionnaire, “1. I am generally satisfied with the workbook homework.” The responses were as follows: very Much (58%), somewhat (30%), undecided (10%), not Really (2%), and not at All (0%) “2. My self-confidence has improved.” The responses were: very much (26%), somewhat (50%), undecided (18%), not really (4%), and not at all (2%). For the question “3. The will to live has increased”, the responses were: very much (36%), somewhat (32%), undecided (30%), not really (0%), or not at all (2%). “4. I have many positive thoughts (hope) and emotions (happiness): very much (32%), somewhat (42%), undecided (20%), not really (4%), and not at all (2%). For “5. It gave me the opportunity to check on my own the problem of not remembering words.”: very much (42%), somewhat (46%), undecided (8%), not really (4%), and not at all (0%). For “6. I am motivated to pursue other studies.”: very much (32%), somewhat (46%), undecided (14%), not really (8%), and not at all (0%). For “7. I think it has helped prevent dementia.”: very much (42%), somewhat (44%), undecided (8%), not really (6%), and not at all (0%).

## Discussion

In this study, self-intervention training for naming using newspaper media was conducted for six weeks (30 sessions) for adults in the pre-clinical stage of dementia. Before and after the intervention, (1) word retrieval, (2) subjective communication, and (3) intervention satisfaction were compared.

The first major result of this study was that in terms of word retrieval, intervention effects were observed in the probe test (fill-in-the-blank) as well as in the confrontation naming (S-K-BNT) and generative naming (semantic and phonemic fluency) tasks. It is thought that the improvement in confrontation naming ability was due to the characteristics of the group related to the cause of difficulty in word retrieval. The SCD group frequently experiences a tip-of-the-tongue phenomenon^24^. If we explain the reason for the tip-of-the-tongue phenomenon based on the transmission deficit hypothesis, adults with SCD have no problem accessing the semantic system. However, the network connecting the semantic system to the phonological system is weakened causing word retrieval failure. In the case of the MCI group, the cause of the decline in word retrieval ability is unclear; howeve, according to general hypotheses, naming defects result from the damage to semantic memory itself^42^, damage to the process of accessing semantic memory rather than damage to semantic memory itself^43^, or damage to the retrieval of appropriate words corresponding to semantic features from the lexical system^44^. The preclinical stage of dementia group in this study explained the correct meaning of the target word during the pre-post tests, or "I can't remember the word quickly, although this meaning should be included here." frequently complained. Therefore, the participants included in this study have difficulty finding and retrieving suitable words from the vocabulary system rather than the loss of semantic memory itself. Therefore, the performance on the confrontation-naming task increased as the semantic-lexical and phonological systems were strengthened while implementing fill-in-the-blank training using context.

The observed intervention effect in the generative naming task may be related to the characteristics of the tasks used in training and the similarity of the cognitive-linguistic processing processes between tasks. Generative naming is a task of retrieving vocabulary corresponding to the presented semantic category (e.g. animals) or exploring vocabulary corresponding to a given phoneme (e.g. ). To solve the 'fill-in-the-blanks", attention is required to read the text to the end, and reasoning is required to grasp the context before and after and infer the appropriate meaning, to enter the blank while utilizing background knowledge. After inferring the meaning, the semantic-lexical system should be activated to find the vocabulary that corresponds to that meaning. In Korean, the postpositional particle behind the word varies depending on the phonetic characteristics of the word (e.g., If there is a final syllable in a word, the nominative particle is '’, and if there is no final syllable, '' is used). Therefore, when selecting the vocabulary, the participants have to consider the postpositional particle after the blank. In addition, to find the most appropriate vocabulary to fill in the blank, it is necessary to approach the semantic-lexical system and repeat the process of exploring and modifying the grammatical form of the vocabulary. In other words, the role of the frontal lobe along with the functions of other lobes can crucial since the executive function that integrates various cognitive subfunctions must be sufficiently activated to perform the 'fill-in-the-blank' task. If the workbook user cannot find an appropriate word to fill in the blank, they will turn over the page and check the semantic and phoneme cues. After recalling the semantic cue in the brain, it goes through the process (phonological-lexical system) of finding a target word using a phoneme cue presented as another cue. In this process, the frontal lobe will be activated since working memory and phonological-lexical systems rely more on word retrieval. For solving the letter completion problem (e.g. Use suggested letters to complete words related to animals) presented as an additional task during the intervention, it is necessary to recall whether the presented word falls under the category of ‘animal,’ look at the phoneme presented, and then search for the ‘animal vocabulary’ that matches the phoneme. This is similar to the process of retrieving appropriate words by using semantic and phonemic clues when words can not be clearly retrieved in the 'fill-in-the-blank' task. An additional task included in the workbook, the initial consonant word task (e.g., write down at least 10 words that begin with '') requires that the process of combining vowels and final consonants with the presented two consonants be repeated to retrieve meaningful words that match the phoneme.

The reason for a greater degree of improvement in the generative naming task than in the confrontation naming task may be due to the difference in cognitive abilities used when performing the two tasks. For confrontation naming, visual information is provided, unlike the workbook fill-in-the-blank training. When trying to name an object, its visual properties are first judged through a visual analysis of the size, shape, and color of the picture. Subsequently, objects are recognized by integrating visual information and matching it with long-term memory. In the semantic system stage for the recognized object, the concept of the use and characteristics of the object stored owing to the accumulation of personal experience or knowledge are used to identify the intrinsic properties of the object. Subsequently, the word in the lexicon and the meaning of the object are connected. At this stage, a specific meaning and appropriate vocabulary are selected^45–47^. Confrontation naming is important for visual recognition and generally depends on linguistic semantic memory. In contrast, the fill-in-the-blanks task included in the workbook requires the ability to generate vocabulary by inferring appropriate meanings without reference and goes through more complex semantic processing than the confrontation naming task. The letter completion task and the initial consonant word task are also related to the activation of the frontal lobe since they involve complex processing and multifaceted cognitive functions such as attention, short-term memory, the phonological-lexical system, and the semantic-lexical system. Therefore, since the tasks included in the workbook consisted of tasks that required more activation of the frontal and temporal lobes in terms of brain function activation, the posterior score may have improved significantly in the generative naming task.

Interestingly, the score of the group that received the intervention improved in the generative naming task, while the score of the group that did not receive the intervention showed lowered pattern in the post-test. These results suggest that for the group that did not receive the intervention, it is possible that they gradually showed difficulty in retrieving words over time. In addition, phonemic fluency improved to a greater extent than semantic fluency within the generative naming task, and differences in the degree of performance improvement were observed according to the subtype. For generative naming, it is known that search strategies and reliance on systems differ depending on subtypes^48^. Phonemic fluency requires a relatively ‘phonological’ search, while semantic fluency requires a familiar ‘semantic’ search. As mentioned above, the group including those in the preclinical stage of dementia generally had no problems accessing the semantic system whereas they faced problems with tasks associated with the network that connects the lexical and phonological systems. Therefore, the score might have improved more in the phonemic fluency task due to the effect of the interventions (letter completion task according to semantic category and word generation task using initial consonants) which strengthened the level of the phonological-lexical system.

The second major finding of this study was that no significant change in subjective communication ability was observed in either group. GICA is a questionnaire composed of items that can evaluate overall communication skills such as ‘attention/memory, language comprehension/production, and communication efficiency’(e.g. ‘I forget what I was trying to say while I was talking,’ ‘It is difficult to understand when I hear long, complex words’). It is possible that the intervention did not have a significant effect on the communication ability experienced in daily life, or that there was no significant difference in the intervention group since the items in the GICA were not directly related to word retrieval.

As the third major finding of this study, a high rate of positive responses was observed in the satisfaction of the participants who received the intervention. The sum of the percentages of positive responses with 'very much’ (5 points) and 'somewhat’ (4 points), is as follows: “‘1. I am generally satisfied with the workbook homework (82.8%)’, ‘2. My self-confidence has improved (74.3%)’, ‘3. The will to live has increased (74.3%,)’, ‘4. Positive thoughts (hope) and emotions (happiness) increased (68.5%)’, ‘5. It gave me an opportunity to check about the problem of not remembering words by myself (85.7%)’, ‘6. I have the motivation to pursue other studies (74.3%)’, ‘7. I think it has helped prevent dementia (85.7%). In addition, workbook users said, “I had nothing to do at home, but I had a good time solving the workbook.”, “My memory seems to improve.”, “It was difficult initially to read newspaper articles, but subsequently, the solving time shortened, and the content of the article became interesting, so I wanted to continue solving it.” The majority of people responded positively to all the items, confirming that they were satisfied not only with the workbook task but also with various aspects of life through the intervention of ‘filling in the blanks in editorial and newspaper articles’.

In summary, improvements in word retrieval abilities were observed after the intervention, and consequently, satisfaction with various aspects of life increased. This is thought to be due to the activation of the frontal and temporal lobes through this intervention, consisting of training to strengthen the lexical-semantic and phonological-vocabulary systems. Until now, no study has examined the word retrieval ability of the cognitive impairment risk group using a fill-in-the-blanks task based on the contents of mass media. Therefore, this is the first intervention study to use a newspaper editorial workbook, and it is noteworthy to investigate the efficacy of word finding through non-face-to-face learning without the restrictions of place and time. Especially, our findings provide the basis for the active application of this intervention method clinically to prevent dementia while showing the possibility of it being used as one of a new therapeutic alternative employing mass communication media in the era of pandemics such as 'COVID-19'.

The limitations of this study were as follows. The study does not investigate SCD and MCI separately. The main purpose of the study was to investigate the effect in pre-dementia cases, and we considered SCD and MCI participants as one group in accordance with the research purpose. To explore the group difference between SCD and MCI, we have already conducted an analysis on 34 SCD participants (17 in both the intervention and control group) and 36 MCI participants (18 in both the intervention and control group) in a preliminary study^49^. As a result, the SCD and MCI groups showed the same patterns of performance; and, there was no significant difference on intervention effect. Since all participants included in the preliminary study were included in the current study, we have focused on analyzing the differences between the intervention and non-intervention groups in the study. In addition, as shown in the Appendix, the SCD and MCI groups did not differ regarding the demographic information except for the MMSE scores. However, the characteristics of the groups might affect the intervention effect. In future research, it is necessary to include participants with dementia and verify the effectiveness of the intervention according to the degree of cognitive decline. At 6 weeks, the duration of the intervention in this study was relatively short, making it difficult to observe the long-term effects and make a direct assessment of dementia progression. Therefore, in future studies, it is necessary to extend the duration of the intervention to confirm the long-term effects and evaluate the progression of dementia through direct assessment of cognitive function. In addition, it is necessary to control the socioeconomic status of research participants and perform large-scale studies in order to increase the possibility of generalization. Since changes in brain function were not confirmed during the workbook training period, it was not possible to exactly determine what kind of cognitive and language processing function the fill-in-the-blanks task required and which brain regions were activated. Therefore, to confirm the usefulness of the fill-in-the-blanks task in follow-up studies, it is necessary to examine word retrieval or information processing through brain imaging studies. In addition, to measure the degree of the subjective appeal on word retrieval ability in the elderly and cognitively impaired groups, it is necessary to develop a scale consisting of questions directly related to word retrieval and confirm changes in subjective word retrieval ability.

### Supplementary Information

Supplementary Tables.

## Data Availability

The data underlying the results presented in this study contain potentially identifying or sensitive patient information and cannot be shared publicly due to restrictions imposed by the Institutional Review Board of Samsung Medical Center (SMC). However, the data are available from the Institutional Review Board of SMC (contact via https://www.e-irb.com:3443/index.jsp) for researchers who meet the criteria for access to confidential data. If the reviewers require anything, the corresponding author (JHY) could be contacted.
