# Supplementary material for: Effects of workbook training using editorials and newspaper articles in adults with preclinical stage of dementia
Source: Sci Rep. 2024 Jan 27;14:2302. doi: 10.1038/s41598-024-52873-z (PMC10821911; doi:10.1038/s41598-024-52873-z)
Supplement: Supplementary file 1 — Supplementary Tables. [file 41598_2024_52873_MOESM1_ESM.docx]

Appendix

| Characteristic | SCD group  (N=50) | MCI group  (N=49) | T |
| --- | --- | --- | --- |
| Age(years) | 71.46(7.09) | 72.22(6.70) | -.551 |
| Education(years) | 13.98(2.93) | 13.84(2.83) | .247 |
| K-MMSE | 28.90(1.31) | 27.24(2.16) | 4.609^***^ |
| SGDS | 2.96(2.46) | 3.02(2.66) | -.117 |

**Table A. Characteristics of SCD and MCI populations****.** K-MMSE: Korean version of Mini-Mental State Examination, MCI: mild cognitive impairment, SCD: subjective cognitive decline, SGDS: Short form Geriatric Depression Scale. *** Significant difference between SCD and MCI group with *p<.05, ** p<.01, *** p<.001.

| Characteristic | SCD group(N=50) | | | MCI group(N=49) | | |
| --- | --- | --- | --- | --- | --- | --- |
|  | intervention group  (N=25) | control group  (N=25) | t | intervention group  (N=25) | control group  (N=24) | t |
| Age(years) | 72.16  (8.19) | 70.76  (5.88) | -.694 | 72.28  (5.53) | 72.17  (7.87) | -.058 |
| Education(years) | 13.96  (3.20) | 14.00  (2.69) | .048 | 13.76  (2.65) | 13.92  (3.06) | .192 |
| K-MMSE | 28.68  (1.70) | 29.12  (.726) | 1.190 | 26.88  (1.87) | 27.63  (2.41) | 1.210 |
| SGDS | 3.48  (2.56) | 2.44  (2.29) | -1.511 | 2.52  (2.31) | 3.54  (2.94) | 1.353 |

**Table B. Characteristics of intervention and control groups in SCD and MCI populations.** K-MMSE: Korean version of Mini-Mental State Examination, MCI: mild cognitive impairment, SCD: subjective cognitive decline, SGDS: Short form Geriatric Depression Scale. *** Significant difference between SCD and MCI group with *p<.05, ** p<.01, *** p<.001.
